# Assessment of Risks and Outcomes of Sinusoidal Obstruction Syndrome/Veno-Occlusive Disease in Allogeneic Stem Cell Transplant Recipients Including Potentially Undiagnosed Cases—A Multicenter Canadian Study

**DOI:** 10.3390/curroncol33050261

**Published:** 2026-04-30

**Authors:** Emily Trus, Alexander Ruzic, Ram Vasudevan Nampoothiri, Gregory R. Pond, Vinita Dhir, Andrew Poskus, Louise Bordeleau, Michael Trus

**Affiliations:** 1 College of Engineering, University of Guelph, Guelph, ON N1G 2W1, Canada; etrus@uoguelph.ca; 2 Department of Graduate Studies, McMaster University, Hamilton, ON L8S 4L8, Canada; ruzica@mcmaster.ca; 3 Department of Medicine, Transplant and Cellular Therapy Program, Ottawa, ON K1H 8L6, Canada; rvasudevan@toh.ca (R.V.N.); anposkus@toh.ca (A.P.); 4 Faculty of Medicine, University of Ottawa, Ottawa, ON K1H 8M5, Canada; 5 Department of Oncology, McMaster University, Hamilton, ON L8V 5C2, Canadabordeleaul@hhsc.ca (L.B.)

**Keywords:** allogeneic stem cell transplant, sinusoidal obstruction syndrome/veno-occlusive disease

## Abstract

**Background and Methods:** Sinusoidal obstruction syndrome/veno-occlusive disease (SOS/VOD) is a severe complication of allogeneic stem cell transplant (allo-SCT). Given the increased use of allo-SCT and variability of SOS/VOD incidence in published reports, cases of allo-SCT from two major transplant centers in Ontario, Canada (2019–2021), were reviewed to identify risk factors prognostic for SOS/VOD onset and to assess outcomes. **Results:** This study included 536 allo-SCT cases, with a mean age of 53.4 years [min–max: 17–76], including 322 male recipients and 214 female recipients. There were 17 SOS/VOD cases diagnosed during the first 100 days, representing 3% of allo-SCT cases, with a median age of 48 years [18–72] and equally distributed between genders. All cases were classical SOS/VOD, with onset prior to day 21 [1–20]. These cases were graded as one mild, six moderate, six severe, and four very severe cases. The mild case of SOS/VOD recovered after treatment with diuretics. In respect to the 16 cases graded as ≥moderate SOS/VOD, the average inpatient stay was 56 days [24–178], and eight patients were in the ICU for an average of 6 days [0–42], with a median of zero days. Five of the sixteen ≥moderate SOS/VOD patients died within 100 days [9–59]—four from SOS/VOD. After day +100, five remained alive, and six died between days 125 and 419. Treatments for ≥moderate SOS/VOD included diuretics [*n* = 15], steroids [*n* = 3], and defibrotide [*n* = 9]. The nine patients treated with defibrotide were graded as moderate [*n* = 2], severe [*n* = 4], and very severe [*n* = 3]. Three of the nine patients treated with defibrotide died before day 100, and the other six survived beyond day 100. None of the six surviving patients died from SOS/VOD. Univariable regression analysis identified a higher baseline absolute neutrophil count (ANC) of 4.2 × 10^9^/L compared to 2.6 × 10^9^/L [*p* = 0.035] and lower baseline platelet count of 104 × 10^9^/L compared to 140 × 10^9^/L [*p* = 0.034] in SOS/VOD and non-SOS/VOD cases, respectively, as independent risks for ≥moderate SOS/VOD. Treatment with inotuzumab ozogamicin was also identified as a risk factor for ≥moderate SOS/VOD (*p* = 0.016). The absence of late-onset SOS/VOD in the cohort of 536 patients prompted a retrospective analysis of the data to identify potentially missed cases. Seven cases were identified as meeting the diagnostic criteria for SOS/VOD: four classical and three late-onset. One case would have been graded as severe, and the remaining six would have been graded as very severe. Six patients were reported to have died between days 11 and 107, with four deaths before day 100. The clinical diagnoses of patients meeting diagnostic criteria for SOS/VOD included infection (*n* = 3), graft-versus-host disease (GVHD) (*n* = 3), and pulmonary hemorrhage (*n* = 1). The inclusion of potentially missed cases in the analysis again suggested a lower baseline platelet count (*p* = 0.002) and prior treatment with inotuzumab ozogamicin (*p* = 0.003) as potential risk factors for SOS/VOD. The baseline ANC was lower in this combined cohort but did not reach statistical significance (*p* = 0.089) as it did in the confirmed SOS/VOD cohort (*p* = 0.035). Additional clinical features that were identified as statistically significant for the onset of SOS/VOD (potential and confirmed cases) included a lower Karnofsky Performance Status (*p* = 0.01), the presence of pulmonary hypertension (*p* = 0.012), lower baseline hemoglobin (*p* = 0.017), and higher baseline serum ferritin (*p* = 0.01). **Conclusions:** The incidence of classical SOS/VOD in this cohort was consistent with recent published reports and carried a high fatality rate. A higher ANC, lower platelet count at the start of the preparative regimen, and prior treatment with inotuzumab ozogamicin were identified as potential risk factors for diagnosed SOS/VOD. Hospital and intensive care unit stays were longer in SOS/VOD patients. There were no cases of late-onset VOD diagnosed within the first 100 days of allo-SCT transplant, which is inconsistent with recently reported incidence rates. Potentially missed cases of SOS/VOD were identified, suggesting that this disease may be under-diagnosed and underscoring the need for ongoing education and resources to allow for early intervention.

## Simple Summary

Sinusoidal obstruction syndrome/veno-occlusive disease (SOS/VOD) is a severe complication of allogeneic stem cell transplant (allo-SCT). Given the increased use of allo-SCT and variability of SOS/VOD incidence in published reports, we reviewed cases of allo-SCT from two major transplant centers in Ontario, Canada (2019–2021), to identify risk factors for SOS/VOD and assess outcomes. We found that the incidence of classical SOS/VOD in the first 21 days after allo-SCT was consistent with recent published reports. Patients with SOS/VOD had longer hospital stays, including intensive care unit treatment, and had a high fatality rate. There were no cases of late-onset VOD diagnosed within the first 100 days of transplant, which differs from recently reported data. We identified potential novel risk factors (higher absolute neutrophil count and lower platelet count) at the beginning of the preparative regimen as well as potential missed cases, highlighting the importance of ongoing surveillance for this disorder.

## 1. Introduction

SOS/VOD is a severe complication of allo-SCT characterized by clinical features of elevated bilirubin, weight gain, ascites, and painful hepatomegaly [1,2]. These clinical characteristics have been used as the basis for the diagnostic criteria developed by the Seattle and Baltimore groups [3,4,5], later refined by the European Society for Blood and Marrow Transplantation (EBMT) [6]. The incidence of SOS/VOD has decreased with the increased use of reduced-intensity conditioning (RIC) regimens, with an early report finding a decrease in incidence from 11.5% to 6.5% over two consecutive decades [7]. The lower incidence of SOS/VOD in later reports remains consistent [8,9,10]. For example, the EBMT working party established a cumulative incidence of classical SOS/VOD of 1.8% in 2886 HSCT recipients, which increased to 2.4% at day 100 [9]. Similar results were found in the Japanese nationwide registry data, where the incidence of classical and late-onset SOS/VOD was reported at 2.5% and 2.2%, respectively [10]. Despite SOS/VOD decreasing in incidence, multiorgan failure (MOF), more recently named multiple organ dysfunction (MOD) [11], continues to be a frequent life-threatening manifestation of SOS/VOD and has been reported in up to 46% of cases in one study [8]. Ruutu et al. reported MOD as the diagnostic criterion for 34 of 35 SOS/VOD cases classified as very severe [9]. Mortality rates of over 80% have been described in SOS/VOD cases presenting with MOD [12,13,14]. Therefore, early recognition is vital to allow for therapeutic intervention because, unabated, the disease has the potential to progress to lethality. Defibrotide is the only available agent with proven efficacy for the treatment of SOS/VOD [15]. An early study noted a decrease in mortality in SOS/VOD cases presenting with MOD over two decades, and the only identifiable factor was treatment with defibrotide [7]. A phase 3 clinical trial documented improved survival at day +100 in SOS/VOD cases with MOD treated with defibrotide compared to rigorously selected historical controls at the established effective and well-tolerated dose of 25 mg/kg/day [16,17]. A number of studies have confirmed the acceptable safety profile of defibrotide and have shown improved survival rates in patients where treatment was given earlier after the diagnosis of SOS/VOD [13,14,15,16,17,18,19,20]. The variability of SOS/VOD incidence reported between international transplant centers [9] and the increasingly frequent use of allo-SCT numbers across Canada [21] prompted the investigators of this study to evaluate the incidence and outcomes of SOS/VOD from two transplant centers in Ontario, Canada (2019–2021), and to identify risk factors prognostic for SOS/VOD onset.

## 2. Methods

This was a retrospective study using institutional databases and electronic medical records from 2019 to 2021 provided by Juravinski Hospital (Hamilton, ON, Canada) and The Ottawa Hospital (Ottawa, ON, Canada) for adult patients (ages ≥ 18) who underwent allo-SCT. The study received local Research Ethics Board approval at both sites. Descriptive statistics were used to summarize patient and treatment characteristics, along with outcomes. Risk stratification for acute myelogenous leukemia (AML) was performed as per the European LeukemiaNet risk stratification [22]. The primary outcome was incidence of SOS/VOD at any time during the transplant course, diagnosed as per the revised EBMT criteria [6]. Potential prognostic factors of SOS/VOD were studied using univariable logistic regression analysis. Due to the small number of patients with SOS/VOD, multivariable models were not constructed. Overall survival estimates for those with SOS/VOD were calculated using the Kaplan–Meier method. All tests were two-sided, and statistical significance was defined as a *p*-value of <0.05. No adjustment was performed for multiple testing, and exact results are presented. The revised EBMT criteria were used for the identification of potentially missed cases of classical and late-onset SOS/VOD [6].

## 3. Results

Data were collected from a total of 536 allogeneic SCT recipients, with 252 cases from Hamilton, ON, Canada, and 284 cases from Ottawa, ON, Canada. The transplant was the first allo-SCT for 497 patients (92.7%) and the second allo-SCT for 39 patients [7.3%].

From the beginning of the preparative regimen to day 100, six patients contracted COVID-19, diagnosed using either a nasopharyngeal swab (NPS) or bronchoscopy. Fifty-one patients [9.5%] died by day 100 [range: 3–100], with no COVID-19-related deaths. There were no deaths before day zero.

In total, there were 167 [31%] documented deaths. The documentation of death beyond day 100 post-transplant was impeded by inconsistent reporting by the referral centers where patients returned for ongoing care.

The demographics of the cohort are summarized in Table 1.

There were 17 diagnosed SOS/VOD cases, representing 3% of SCT cases, with a mean age of 49.6 years [18–72], comprising nine male recipients and eight female recipients. Seventy-six percent [*n* = 13] were older than 40 years, and all were early-onset prior to day 21 and had a diagnosis made on average at day 11 [3–20], with one mild, six moderate, six severe, and four very severe cases. Transplant indications included AML [*n* = 5], acute lymphoblastic leukemia (ALL) [*n* = 4], MDS [*n* = 2], secondary AML [*n* = 2], and primary myelofibrosis (PMF) [*n* = 2], with one patient with chronic myelogenous leukemia (CML) [6%] and another patient with MPN—other [6%]. The ELN risk value was reported in all five cases of AML and comprised adverse [*n* = 4, 80%]- and intermediate [*n* = 1, 20%]-risk patients. Two patients were treated with inotuzumab ozogamicin pre-transplant, and no patient received treatment with gemtuzumab ozogamicin.

The remission statuses for patients at the time of allo-SCT were CR [*n* = 12, 71%], no response [*n* = 4, 23%], and never treated [*n* = 1, 6%]. The KPS for the SOS/VOD cohort included scores of 100% [*n* = 1, 5.9%], 90% [*n* = 2, 11.8%], 80% [*n* = 9, 52.9%], and 70% [*n* = 5, 29.4%]. Only eighteen percent of the SOS/VOD cohort scored 90% or higher compared to thirty-eight percent of the entire cohort.

Preparative regimen data were available for all VOD patients: the majority received a myeloablative regimen [*n* = 13, 76.5%], and the remainder received an RIC regimen [*n* = 4, 23.5%]. Donor types were MRD [*n* = 7, 41.2%] and MUD [*n* = 10, 58.8%]. No SOS/VOD cases were reported in the cohort of patients with a haploidentical donor type.

Specific preparative regimen data for this cohort included FLU BU4 [*n* = 7, 41.2%], Cy TBI [*n* = 4, 23.5%], FLU BU2 [*n* = 2, 11.8%], BU Cy [*n* = 2, 11.8%], FLU Mel [*n* = 1, 5.9%], and the Kroger protocol [*n* = 1, 5.9%]. IV busulfan administration was slightly higher in the SOS/VOD cases, where seventy-one percent of patients [*n* = 12] received this agent compared to sixty-two percent of the entire cohort.

Pre-existing hepatic abnormalities were reported in six of the confirmed SOS/VOD cases (35%), equally distributed between cases of unexplained transaminitis and fatty liver. In comparison, pre-existing hepatic abnormalities were documented in twenty-five percent [*n* = 113 (21%)] of the non-SOS/VOD cohort. Prior cholecystectomy was rare [*n* = 1, 6%] and occurred remotely, and no patient had a prior splenectomy. There were no cases of COVID-19 among any of the patients diagnosed with SOS/VOD.

All patients [100%] met the criterion for bilirubin increase, with an average value of 186 micromole/L [37–956], and the average day this occurred after day zero was day 10. Additionally, seven [41.2%] patients met the hepatomegaly criterion, and this criterion on average was documented nine days post day zero. A five percent weight gain occurred in twelve [70.6%] cases at an average of 10 days post day zero. The average increase in weight in all SOS/VOD cases above baseline was 9.4 kg [1–30]. Ascites was reported in thirteen patients [76.5%] at an average of 10 days post day zero.

The one mild case of SOS/VOD was resolved with the administration of a diuretic, and the patient survived beyond day +100. Risk factors for the development of ≥moderate SOS/VOD were analyzed using logistic regression models (Supplementary Materials Table S1). Univariable regression analysis identified a higher baseline ANC of 4.2 × 10^9^/L compared to 2.6 × 10^9^/L [*p* = 0.035] and a lower platelet count of 104 × 10^9^/L compared to 140 × 10^9^/L [*p* = 0.034] in SOS/VOD and non-SOS/VOD cases, respectively, as independent risk factors for developing ≥moderate SOS/VOD.

For ≥moderate SOS/VOD, the average inpatient stay was 56 days [24–178] compared to 36 days in the non-SOS/VOD cohort, and eight patients were in the ICU for an average of 6 days [0–42], with a median of zero days for all diagnosed SOS/VOD cases, compared to a 1 day average and median of zero days in the non-SOS/VOD cohort.

The demographics of the entire cohort and of SOS/VOD patients presenting with ≥moderate disease are shown in Table 1.

**Figure 1:**
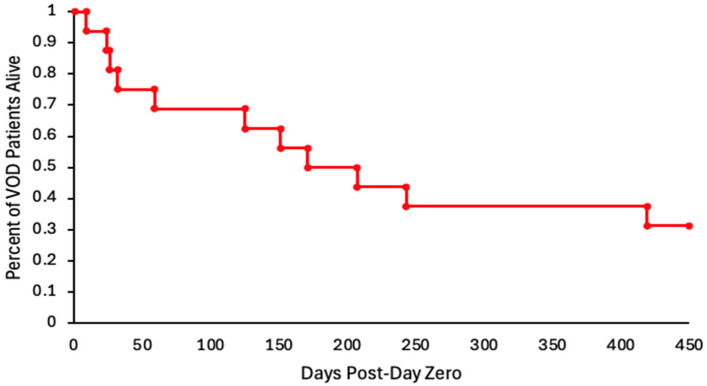
Kaplan–Meier estimate of overall survival of patients with ≥moderate SOS/VOD after day zero post allo-SCT.

Five of the seventeen confirmed SOS/VOD patients died within 100 days [9–59], with four dying from SOS/VOD. Five remained alive at the time of writing this paper, and six died after day 100 [125–419]. Treatments for SOS/VOD included diuretics [*n* = 15], steroids [*n* = 3], and defibrotide [*n* = 9]. The average number of defibrotide doses received was forty-six. The nine patients treated with defibrotide were graded as moderate [*n* = 2], severe [*n* = 4], and very severe [*n* = 3], with seven dying overall and three dying before day 100. Therefore, sixty-seven percent of the SOS/VOD patients treated with defibrotide survived beyond day 100, consistent with studies confirming the efficacy of this agent in the management of SOS/VOD [15,16,17]. Of the eleven SOS/VOD patients who survived beyond day 100, five remained alive and six died on average 133 days after transplant [125–419]. After day 100, death occurred due to relapse of the original disease [*n* = 2], sepsis [*n* = 2], graft-versus-host disease [*n* = 1], and graft failure [*n* = 1]. Although twenty-five percent of SOS/VOD patients died from a transplant-related complication, death from SOS/VOD occurred in less than one percent of the entire cohort. The overall survival of SOS/VOD cases is shown in Figure 1.

The absence of late-onset SOS/VOD cases prompted a detailed retrospective review of the data to determine if there were potentially missed cases of SOS/VOD. In retrospect, there were seven cases that met the diagnostic criteria for SOS/VOD, including four potentially missed cases of classical SOS/VOD and three cases of potentially missed late-onset SOS/VOD. One case would have been graded as severe, and the remaining six would have been graded as very severe. All potentially missed cases of SOS/VOD met the diagnostic criteria for classical SOS/VOD, while none of the potential late-onset cases met the diagnostic criteria, based on imaging modalities or histology [6,15]. As expected, defibrotide was not prescribed for any of the potentially missed cases of SOS/VOD. Six patients were reported to have died between days 11 and 107 post SCT with four deaths before day 100. The clinical diagnoses for these patients who met the diagnostic criteria for SOS/VOD included infection (*n* = 3), GVH (*n* = 3), and pulmonary hemorrhage (*n* = 1). The characteristics of patients identified as potentially missed SOS/VOD cases are summarized in Table 2.

The potentially missed cases and previously diagnosed cases were analyzed together to identify potential risk factors for SOS/VOD. Similar to the analysis that assessed confirmed SOS/VOD cases, a lower baseline platelet count at the beginning of the preparative regimen (*p* = 0.002) was identified through univariable regression analysis as a potential risk factor for SOS/VOD. The baseline ANC in this combined cohort did not reach statistical significance (*p* = 0.089), as it did in the confirmed SOS/VOD cohort (*p* = 0.035). Additional clinical features that were identified as statistically significant in the combined cohort of SOS/VOD (potential and confirmed cases) included previous treatment with inotuzumab ozogamicin (*p* = 0.003), lower KPS (*p* = 0.01), the presence of pulmonary hypertension (*p* = 0.012), lower baseline hemoglobin (*p* = 0.017), and higher baseline ferritin (*p* = 0.01). The results are summarized in Table 3.

## 4. Discussion

The incorporation of allo-SCT into treatment strategies for hematological malignancies has steadily increased over the years [23], and this trend has also been observed in Canada, where allogeneic HSCT increased by 22.3% over two separate decades [21]. SOS/VOD, a potentially life-threatening complication of allo-SCT, has fortunately decreased in incidence, attributed in part to the increased use of RIC regimens [7,8,9,10]. Given the expansion of allo-SCT and variability in the incidence of SOS/VOD in various reports, we aimed to assess the incidence and outcomes of SOS/VOD at two Canadian transplant centers and identify risk factors for SOS/VOD. We also analyzed whether a strict application of the criteria could detect potentially missed cases.

Consistent with other studies, the incidence of SOS/VOD was low in this cohort, with only 17 cases diagnosed, representing 3% of patients. There were one mild case and six moderate cases of SOS/VOD in the cohort, while the remaining cases were graded as severe and very severe. The finding that SOS/VOD presents more commonly with ≥moderate disease is consistent with the results reported by Ruutu et al., where the severity grade was reported in 67 of 93 SOS/VOD cases from 2886 allogeneic SCT patients and over 90 percent of cases were graded as severe or very severe [9]. UDCA, an agent that has been shown to reduce hepatotoxicity post allo-SCT, was administered prophylactically to 93% [*n* = 499] of the patients in this cohort [24]. However, defibrotide, which has been shown to reduce the incidence of SOS/VOD, was not administered prophylactically to any of the 536 SCT patients in this cohort [25,26].

A number of established modifiable and non-modifiable risk factors for SOS/VOD have been identified, including the intensity of the conditioning regimen [11,27]. The reduction in SOS/VOD incidence can be attributed to the increased use of RIC regimens [7]. Our data are in line with other studies, as the majority of SOS/VOD cases received an MA conditioning regimen (11/17), representing 76% of the SOS/VOD cohort, while MA regimens were used in 48% of the entire cohort. The other four SOS/VOD cases received RIC preparative regimens; this measured incidence of 1.4% is at the lower end of previously reported incidence rates. Interestingly, no cases of SOS/VOD were reported in the 61 patients who underwent a haploidentical transplant using a standard conditioning regimen, compared to the 5.8% incidence of VOD reported in 797 haplo-HSCT patients from nine centers in Spain [28,29]. Age, gender, unrelated donor source, pre-existing liver diseases (elevated transaminases, fatty liver, idiopathic hepatitis, cirrhosis, hepatomegaly, drug toxicity, and viral hepatitis), acute leukemia, second transplant, and elevated ferritin levels, which are known risk factors for SOS/VOD, were not identified as risk factors in the cohort diagnosed with SOS/VOD during the post-transplant period [27]. A KPS of less than 90, an established risk factor for SOS/VOD, was documented in 81% of the VOD cohort compared to 62% of the entire cohort, but this difference did not reach statistical significance [27]. There was a trend in AML patients who developed SOS/VOD having adverse ELN scores (80%) compared to AML patients that did not develop SOS/VOD (37%). As of this study, a higher baseline ANC at the beginning of the preparative regimen has not been previously reported as a risk factor for SOS/VOD (*p* = 0.035). However, both early ANC engraftment and an elevated white blood cell count have been reported as risk factors for VOD/SOS in patients undergoing allo-SCT [27,30]. A lower baseline platelet count at the onset of the conditioning regimen was identified as a risk factor for SOS/VOD in this cohort (*p* = 0.034). Interestingly, although thrombocytopenia has not been identified as a risk factor for SOS/VOD in adults, unexplained transfusion/refractory thrombocytopenia is included within the diagnostic criteria in pediatric patients [31]. Baseline ANC and platelet counts were not evaluated as potential risk factors for SOS/VOD in two large studies: a study of Japanese registry data including 16,518 allo-SCT patients and a large meta-analysis of 27,279 patients [10,32]. The high baseline ANC and low platelet count identified as risk factors in our study could be a reflection of the presence of other established risk factors for SOS/VOD, including low KPS, liver disease, and the toxicity of prior therapies [27]. The identification of a higher baseline ANC and lower platelet count is in keeping with the known pathophysiology of SOS/VOD, including the known proinflammatory effects of neutrophils and the procoagulant state that develops early during disease onset [33,34]. A recent study demonstrated that plasma from patients undergoing allo-SCT had a higher capacity to trigger the release of extracellular traps that mediated inflammation compared to healthy controls, and this finding was correlated with the Endothelial Activation and Stress Index (EASIX) [35]. The high baseline ANC in our study could be a potential surrogate for serum lactate dehydrogenase and could, in combination with lower baseline platelets, be a reflection of higher EASIX scores, a known predictor of SOS/VOD [36]. This study has a number of limitations, including its retrospective design, small sample size for the outcome of interest, and insufficient power to conduct multivariable models.

The absence of late-onset SOS/VOD cases in our cohort is inconsistent with other reports showing incidence rates in large transplant cohorts of 2.2–2.4% [9,10]. Other centers have reported the issue of potentially missed cases of SOS/VOD, with EBMT registry data identifying, retrospectively, SOS/VOD as a potential cause of MOF/MOD leading to death in 48 of 202 allo-SCT recipients [37]. The potentially missed cases in our cohort were critically ill, with other conditions potentially mimicking SOS/VOD [27]. This is a likely explanation for the alternative diagnoses other than SOS/VOD made in this group. By including potentially missed cases of SOS/VOD in the analysis along with all diagnosed SOS/VOD cases, we found that the baseline ANC in SOS/VOD was no longer significantly different from the entire cohort. However, the baseline platelet count remained significantly lower in the SOS/VOD cohort (*p* = 0.002), along with treatment with inotuzumab ozogamicin (*p* = 0.003). Baseline hemoglobin was found to be lower in the composite SOS/VOD population (*p* = 0.017), and pulmonary hypertension was more frequently found pre-transplant compared to the entire cohort (*p* = 0.012). The inclusion of potentially missed SOS/VOD cases resulted in the emergence of established risks for SOS/VOD, such as higher ferritin levels (*p* = 0.007) and lower KPS scores (*p* = 0.01) [27].

As with other studies, SOS/VOD was more likely to present with advanced disease [8,9]. The economic burden of this disease has been previously documented [8] and is reflected in our study, where diagnosed VOD cases had longer hospitalizations, including more days spent in the ICU compared to non-SOS/VOD patients. Defibrotide was an effective agent, where 67% of treated patients survived to day 100. It is unknown whether defibrotide treatment would have made an impact on the 86% incidence of mortality in the group of potentially missed SOS/VOD patients.

## 5. Conclusions

In our study cohort, the incidence of classical SOS/VOD cases was similar to other reports and carried a high fatality rate. A higher ANC and lower platelet count at the start of the preparative regimen were identified as potential novel risk factors for SOS/VOD. Hospital and ICU stays were longer in SOS/VOD patients, who were more likely to die within the first 100 days after allo-SCT. Sixty-seven percent of patients treated with defibrotide survived beyond day 100. Furthermore, late-onset SOS/VOD was likely under-diagnosed in this cohort, underscoring the need for a strict application of diagnostic criteria and ongoing education and resources to allow for early intervention.

## Figures and Tables

**Table 1:** Demographics of the entire cohort of allo-SCT patients and SOS/VOD patients presenting with ≥moderate disease as per EBMT severity grading. UDCA = ursodeoxycholic acid, hgb = hemoglobin, MRD = matched related donor, MUD = matched unrelated donor, MA = myeloablative, RIC = reduced-intensity conditioning, NMSCT = non-myeloablative stem cell transplant.

	Overall (*n* = 536)	≥Moderate VOD (*n* = 16)
Age (Years; mean)	53.4	49
Age (>40 Years)	436 (81%)	12 (75%)
Sex		
Female	214 (40%)	8 (50%)
Male	322 (60%)	8 (50%)
KPS < 90%	329 (62%)	13 (81%)
Diagnosis		
AML	172 (32%)	5 (31%)
ALL	76 (14%)	3 (19%)
MDS	86 (16%)	2 (12.5%)
Secondary AML	49 (9%)	2 (12.5%)
PMF	33 (6%)	2 (12.5%)
CML	11 (2%)	1 (6.3%)
MPN—Other	6 (1%)	1 (6.3%)
Lymphoma	65 (12%)	0
Other	38 (7%)	0
AML ELN Risk (*n* = 193 Reported)		
Favorable	36 (19%)	0
Intermediate	83 (43%)	1 (20%)
Adverse	74 (38%)	4 (80%)
Complete Remission	336 (63%)	11 (68%)
Pre-Existing Hepatic Abnormality	113 (21%)	5 (31%)
Inotuzumab	12 (2%)	2 (13%)
Gemtuzumab	5 (1%)	0
Baseline		
ANC (×10^9^/L)	2.6	4.2
Hgb (g/L)	105	93
Platelet (×10^9^/L)	140	104
Ferritin (µg/L)	909	1228
>First BMT	39 (7%)	0
Donor Type		
MRD	114 (21%)	6 (37.5%)
MUD	365 (68%)	10 (62.55)
Haploidentical	57 (11%)	0
Conditioning Intensity		
MA	256 (48%)	12 (75%)
RIC	241 (44.9%)	4 (25%)
NMSCT	38 (7.1)	0
Busulfan-Based Regimen	332 (62%)	12 (75%)
TBI Administered	156 (29%)	3 (19%)
UDCA Treatment	499 (93%)	15 (94%)
Weight Change at Day Zero	−0.4 kg	+0.4 kg
Hospital Days (Average)	37	56
ICU Days (Median)	0	0
Deaths by Day 100	51 (9.5%)	5 (31%)

**Table 2:** Demographics and outcomes of potentially missed cases that met diagnostic criteria for SOS/VOD following allo-SCT.

	Age (Years)	Sex	Transplant Indication	First or Second HSCT	Regimen Intensity	Donor Source	Onset	Diagnosis (d)	Severity Grading (EBMT)	Outcome	Presumed Diagnosis
Case 1	49.8	M	Loss of Graft	Second	RIC	MUD	Early	9	Very Severe	Died (d11)	Pneumonia
Case 2	57.4	M	AML	Second	RIC	MUD	Early	7	Very Severe	Alive (d100)	Sepsis
Case 3	72.8	F	ALL	First	RIC	MUD	Late	38	Very Severe	Died (d62)	GVHD
Case 4	40.1	F	MDS	First	MA	MUD	Late	105	Very Severe	Died (d107)	IFI/GVHD
Case 5	67.2	M	CLL	First	Haplo	MRD	Early	14	Very Severe	Died (d19)	Sepsis
Case 6	64.3	M	MDS	First	RIC	MUD	Late	88	Severe	Died (d106)	GVHD
Case 7	45.6	F	PMF	First	RIC	MUD	Early	15	Very Severe	Died (d19)	Pulmonary Hemorrhage

GVHD, graft-versus-host disease; IFI, Invasive Fungal Infection; CLL, chronic lymphocytic leukemia.

**Table 3:** Incidence of previously published and novel risk factors identified in our cohort (ANC and platelet count at baseline) in diagnosed and potentially undiagnosed SOS/VOD patients, and results of univariable logistic regression analysis.

	≥Moderate VOD (*n* = 16)	*p*-Value	SOS/VOD—Potential and Diagnosed (*n* = 24)	*p*-Value
Age (>40 Years)	12 (75%)	-	20 (83%)	-
KPS < 90%	13 (81%)	-	20 (83%)	0.002
AML	5 (31%)	-	7 (29%)	-
ALL	3 (19%)	-	5 (21%)	-
Secondary AML	2 (12.5%)	-	2 (8%)	-
Pre-Existing Hepatic Abnormality	5 (31%)	-	8 (33%)	-
Inotuzumab	2 (13%)	0.016	3 (13%)	0.003
Gemtuzumab	0	-	0	-
Baseline				
ANC (×10^9^/L)	4.2	0.035	1.8	-
Hgb (g/L)	93	-	88	0.017
Platelet (×10^9^/L)	104	0.034	89	0.002
Ferritin (µg/L)	1228	-	3520	0.01
Pulmonary Hypertension	1 (6%)	-	3 (13%)	0.012
>first allo-SCT	0	-	3 (12%)	-
Deaths by Day 100	5 (31%)	-	9 (38%)	-

## Data Availability

The original contributions presented in this study are included in the article. Further inquiries can be directed to the corresponding author.

## Supplementary Materials

The following supporting information can be downloaded at: https://www.mdpi.com/article/10.3390/curroncol33050261/s1. Table S1. Variables included in the univariable regression analysis. Baseline defined as the day the SCT preparative started.
