# Supplementary material for: Assessment of Risks and Outcomes of Sinusoidal Obstruction Syndrome/Veno-Occlusive Disease in Allogeneic Stem Cell Transplant Recipients Including Potentially Undiagnosed Cases—A Multicenter Canadian Study
Source: Curr Oncol. 2026 Apr 30;33(5):261. doi: 10.3390/curroncol33050261 (PMC13205504; doi:10.3390/curroncol33050261)
Supplement: Supplementary file 1 [file curroncol-33-00261-s001.zip › curroncol-4205679-supplementary.pdf]

Supplementary File

Table S1. Variables included in the univariable regression analysis. Baseline defined as the day the SCT preparative started.

**Characteristic**	**Comparator**
Age at SCT	Year
Sex	M vs. F
Principal Diagnosis	N (%)<br>AML Primary<br>ALL<br>CML<br>MDS<br>PMF<br>MPD other<br>Secondary AML
Remission Status at Transplant	N (%)<br>Complete<br>No Response<br>Never Treated
Prior Inotuzamab Treatment	Y vs. N
Prior Gemtuzamab Treatment	Y vs. N
Preexisting Liver Abnormality	Y vs. N
Viral Hepatitis	No<br>HepA<br>HepB<br>HepC
Prior Cholecystectomy	Y vs. N
Prior Splenectomy	Y vs. N
Pulmonary Hypertension	Y vs. N
Congestive Heart Failure	Y vs. N
Iron Reduction Therapy	Y vs. N
Liver/Abdominal Irradiation	Y vs. N
Second BMT	Y vs. N
Ursodeoxycholic Treatment	Y vs. N
Left Ventricular Ejection Fraction	unit
Weight Change (baseline to day 0)	kg
Weight Change (admission to day 0)	kg
Body Mass Index (BMI)	unit
HCT-CI Score	score
Absolute Neutrophil Count (baseline)	unit
Hemoglobin (baseline)	unit
Platelet Count (baseline)	unit
AST (baseline)	unit
ALT (baseline)	unit
Bilirubin (baseline)	unit
Albumin (baseline)	unit
Serum Creatinine (baseline)	unit
eGFR (baseline)	unit
Ferritin (baseline)	unit
Karnofsky Performance Scale Index (KPS)	per 10 units
